# Localisation of Bladder Incontinence Based on History and Examination for First-Year Neurosurgical Residents

**DOI:** 10.21315/mjms-03-2026-185

**Published:** 2026-04-30

**Authors:** Ismail Junaih Shareef, Jafri Malin Abdullah, Zaitun Zakaria, Wing Hang Yop, Mohamad Haikal Zainol, Fadhli Suliman, Azwa Maulat Kamarul Bahri, Aaron Lee Zhi Wen, Xin Yi Jap, Muhammad Zakwan Zulkifli, Muhammad Afifi Shukri Soo

**Affiliations:** 1 Department of Neurosciences, School of Medical Sciences, Universiti Sains Malaysia, Health Campus, Kubang Kerian, Kelantan, Malaysia; 2 Brain and Behaviour Cluster, School of Medical Sciences, Universiti Sains Malaysia, Health Campus, Kubang Kerian, Kelantan, Malaysia; 3 Department of Neurosciences and Brain Behaviour Cluster, Hospital Universiti Sains Malaysia, Universiti Sains Malaysia Specialist Hospital, Health Campus, Kubang Kerian, Kelantan, Malaysia

**Keywords:** localisation, history taking, clinical examination, urinary incontinence, neurology

## Abstract

When a patient presents with urinary incontinence, the goal is to quickly localise the cause of it, so that further imaging workup and targeted management can be initiated at the earliest. In this review, we look at established functional magnetic resonance imaging data and activation likelihood estimation analysis to pinpoint specific areas of the nervous system causing urinary incontinence. These can range from the cortex and the pontine micturition centre to the spinal pathway and peripheral nerves. By using basic history-taking and examination techniques, the five main types of urinary incontinence can be localised to specific anatomical locations.

## Introduction

Urinary incontinence is defined as the involuntary passage of urine (1). The neuroanatomical control of micturition involves a complex hierarchy spanning the prefrontal cortex, brainstem and spinal cord. It is notoriously difficult for neurosurgical residents to accurately localise the source of involuntary urine passage in a clinical setting due to the vast range of possible lesions, often presenting with overlapping symptoms.

This article aims to simplify this process by providing a structured diagnostic process that is easy to carry out in a clinical setting or at the bedside to facilitate diagnosis, localisation of lesions and further focused investigations and management planning. This approach can help shorten the time until diagnosis and eventual management, reducing costs and sparing limited imaging resources. The process of history taking and examination is presented here in the form of flowcharts, which can be easily followed while evaluating patients.

## Types of Urinary Incontinence

There are five main types of urinary incontinence, all caused by distinct lesions in the nervous system, as explained below (2): stress incontinence, urge incontinence, overflow incontinence, mixed incontinence and functional incontinence (1). Stress incontinence is caused by a sphincter that fails to maintain a seal against increased intra-abdominal pressure, which is characterised by the loss of urine during physical activities (3). Urge incontinence usually results from overactivity of the detrusor muscle, causing uninhibited or involuntary bladder contractions during the filling phase (4). Overflow incontinence occurs after the bladder becomes overdistended due to an inability to empty properly, usually presenting with constant dribbling, hesitancy and reduced urge to urinate despite a full bladder (5). Mixed incontinence involves a combination of stress and urge incontinence (1). Finally, functional incontinence is caused by extragenitourinary factors, specifically cognitive deficits or physical impediments that prevent the patient from reaching a toilet in time (1).

## Anatomical Basis of Neurourology

Lower urinary tract (LUT) innervation is schematically represented by the pudendal, pelvic and hypogastric nerves connecting the bladder and urethral sphincter to the spinal cord. From there, the nerve signals reach the brain and form a complex network. This supra-spinal control of the LUT has historically been described in many different ways, including via cadaver dissection and animal models, while the use of functional magnetic resonance imaging (fMRI) for this purpose has recently become popular (6). To achieve imaging, fMRI relies on the association of neuronal activity with the increases in blood flow and oxygen demand that occur when neurons are recruited, also known as the measured blood level oxygen dependence (BOLD) signal. In the field of neurourology, the process of micturition leads to an increase in neuronal activity, which can be detected by fMRI (6).

Two different modalities are typically employed to obtain neurourological insights using fMRI. The first involves a resting state, and the second involves task-related acquisition. For scans performed in a resting state, the acquisition time can be extended, serving as a baseline representing quiescent activity in the region of interest. In the context of analyses of incontinence, separate scans can be performed for image acquisition of the discrete (empty and full) phases of the micturition cycle. Task-related acquisitions accelerate the filling/emptying process to ensure that a full cycle is repeated many times in a single fMRI session. Tasks may also include external physical stimuli to promote the sensation of a full bladder. However, this second modality involves a more invasive preparation than the first. For example, the patient’s bladder can be filled and drained via a catheter to reproduce an accelerated micturition cycle, as is the case in urodynamic testing (7).

Functional neuroimaging is a powerful and versatile tool for investigating the neural structures and processes involved in central LUT control. LUT motor control is usually considered to include storage and micturition (detrusor control as an autonomous activity), but descending LUT control also includes voluntary, conscious pelvic floor muscle contractions (PFMCs). Voluntary PFMCs are a proxy for involuntary tonic contractions employed during the storage phase. In fact, PFMCs tend to be a voluntary “backup” mechanism that is employed during a very strong urge to void or defecate (8).

### The Process of Micturition

In the brainstem, the periaqueductal grey area (PAG) acts as a region for integrating incoming central nervous system messaging (cerebrum, cerebellum, brainstem and spinal cord). When the excitatory signal reaches a sufficient level, it triggers activation of the pontine micturition centre (PMC), initiating the micturition cycle.

The PMC, via one long descending pathway to the sacral spinal cord, controls both the relaxation of the external urinary sphincter and the contraction of the detrusor, although micturition in healthy humans relies on this reflex circuitry only during early infancy. During brain maturation and toilet training, this reflex is gradually put under suprapontine control (8).

Connected to the PMC and PAG are the thalamus and hypothalamus, which are involved in the subsequent phase of micturition. Finally, the most complex stage of micturition involves several regions of the cortical grey matter, notably the insula, prefrontal cortex, anterior cingulate cortex, parahippocampal cortex, supplementary motor area and cerebellum.

An fMRI study by Michels et al. (7) illustrates in Figure 1, activation patterns in 22 healthy males during micturition.

The functional experiment consisted of two blocks presented in a random order during a 300 s scanning session. Block 1 comprised two conditions: REST (visual fixation) and IMITATE (subjects visually imagine starting micturition). Block 2 comprised four conditions: REST (visual fixation), INITIATE (subjects should start micturition), URINATE (actual micturition; urine is flowing) and STOP (interruption of micturition). The onset of each of the conditions REST, IMITATE, INITIATE, and STOP was defined as the moment the particular visual cues for the respective conditions were presented, while the onset of URINATE was determined by the actual onset of micturition; that is, urine flow was detected by the flow detector.

During IMITATE, both groups of subjects showed activity in the middle frontal gyrus (MFG), temporal and cerebellar areas. Activity in the inferior frontal gyrus (IFG) was detected only in voiders, while activity in the insular cortex and supramarginal gyrus (SMG) was detected only in non-voiders.

During INITIATE-E (early), voiders demonstrated significant bilateral activity in the IFG, as well as activities in the right insula and the periaqueductal grey area (PAG). In contrast, non-voiders demonstrated significant activity only in the cerebellum. During INITIATE-L (late), the following were noted (Figure 2):

- Voiders demonstrated significant increases in BOLD signal in parietal opercula (OP) 1 and 4, bilateral MFG, left cerebellum, left thalamus (ventral posterolateral nucleus), left middle temporal gyrus (MTG), left precentral gyrus, right posterior cingulate cortex (PCC), right precuneus and right pons.
- In contrast, non-voiders showed significant increases in BOLD signal in the superior temporal gyrus and inferior parietal lobe bilaterally, left OP 4, left parahippocampal gyrus, left anterior midcingulate cortex (aMCC), right posterior midcingulate cortex (pMCC), right PCC, right superior frontal gyrus, right MTG and right SMG.
- A comparison of voiders and non-voiders revealed voider-specific increases in BOLD signal in the right PCC and pMCC, right OP 1 and OP 4, right PAG, left cerebellum and pons.

During URINATE, voiders demonstrated significant activity only in the cerebellum. Interrupting micturition (STOP condition) in voiders was associated with significant activation in the PAG, anterior cingulate cortex (ACC) and cerebellum. The results of this study provide various novel insights into the control of micturition. First, in voiders, supraspinal activity, including in the pons, is most prominent just before micturition and subsides once actual micturition has started. Second, initiation of micturition in voiders induces significant activity in the brainstem, insula, thalamus, PFC, OP and cingulate cortex. Third, inter-regional coupling is significantly stronger during the initiation of micturition than during actual micturition, especially among the OP, MFG and thalamus (8).

A systematic review by Groenendijk et al. (9) in 2021 summarised the existing evidence on supraspinal motor control of the LUT in humans (i.e. micturition and PFMCs) and on efforts to explore the core brain areas involved in these functions using activation likelihood estimation (ALE) analysis. The study conducted a coordinate-based meta-analysis of existing neuroimaging data to achieve a comprehensive overview of the relevant brain regions involved. ALE analysis was then performed to determine the statistical probability of the brain regions being consistently activated during a specific task.

For PFMCs, ALE analysis of 133 peak coordinates derived from 12 different studies with 74 men and 95 women yielded 10 active clusters using a statistical threshold of *P* = 0.001, uncorrected, with a minimal cluster size of 100 mm^3^. Under these conditions, the ALE clusters included the primary motor cortex, SMA, cingulate gyrus, insula, thalamus, substantia nigra/red nucleus and cerebellum (8) (Figure 3).

Meanwhile, for micturition, ALE analyses of 98 peak coordinates derived from eight different studies with a total of 107 subjects (48 men and 59 women) yielded seven active peak activations/clusters, using a statistical threshold of *P* = 0.001 uncorrected with a minimal cluster size of 100 mm^3^. These ALE clusters included the PMC, PAG, thalamus, cingulate gyrus, frontal gyrus and insula. Cluster 1 is a merged cluster, showing multiple peak activations in the thalamus, PMC and PAG (8) (Figure 4).

## Approach to Diagnosis: History-taking

**Figure 6:**
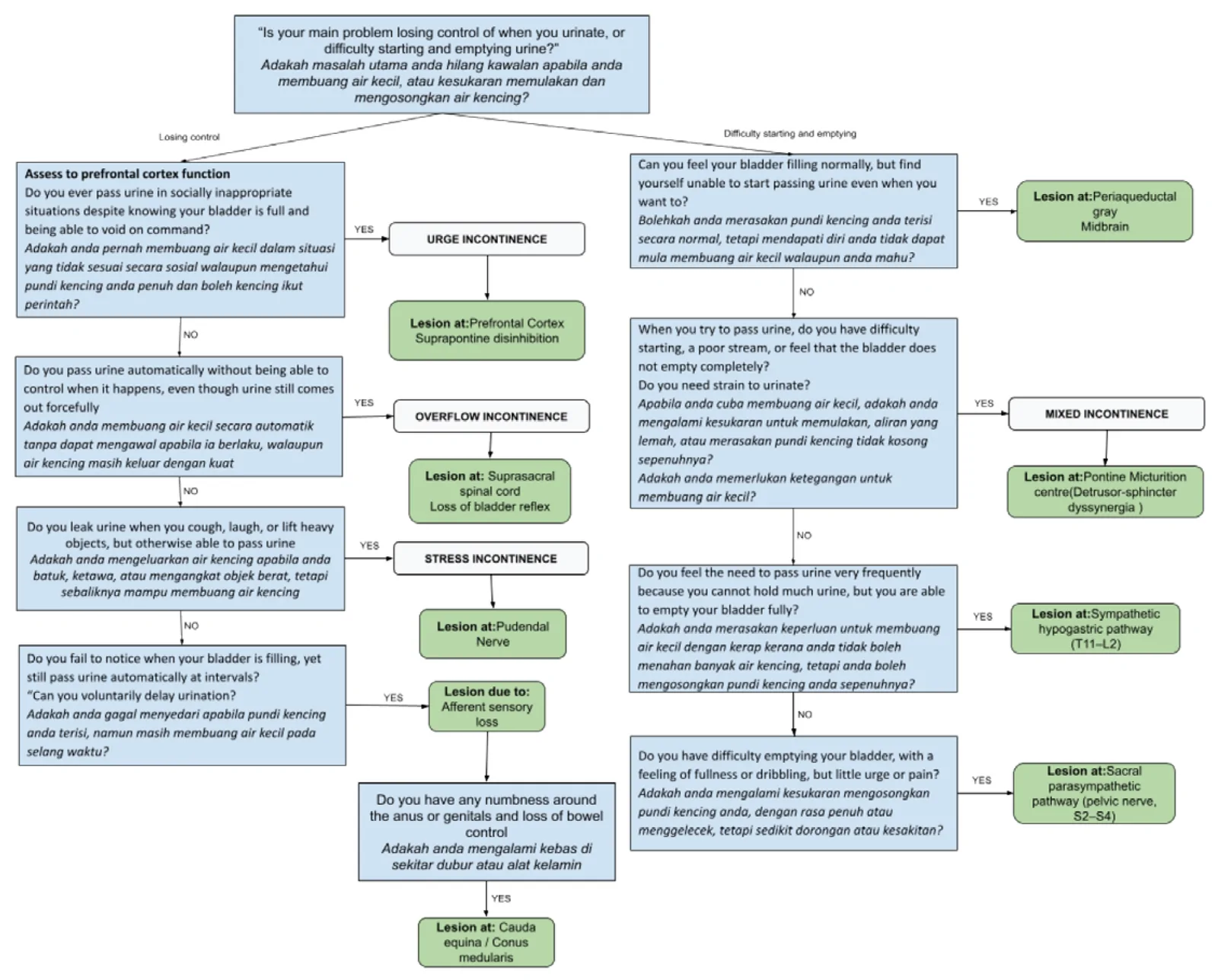
Flowchart for history taking

### Additional History-taking for Localisation

Table 1 above shows a diagram depicting the anatomical locations of structures involved in micturition including Figures 5–7 which shows how history taking and physical examination can localise the cause of incontinence.

## Approach to Diagnosis: Examination

**Figure 7:**
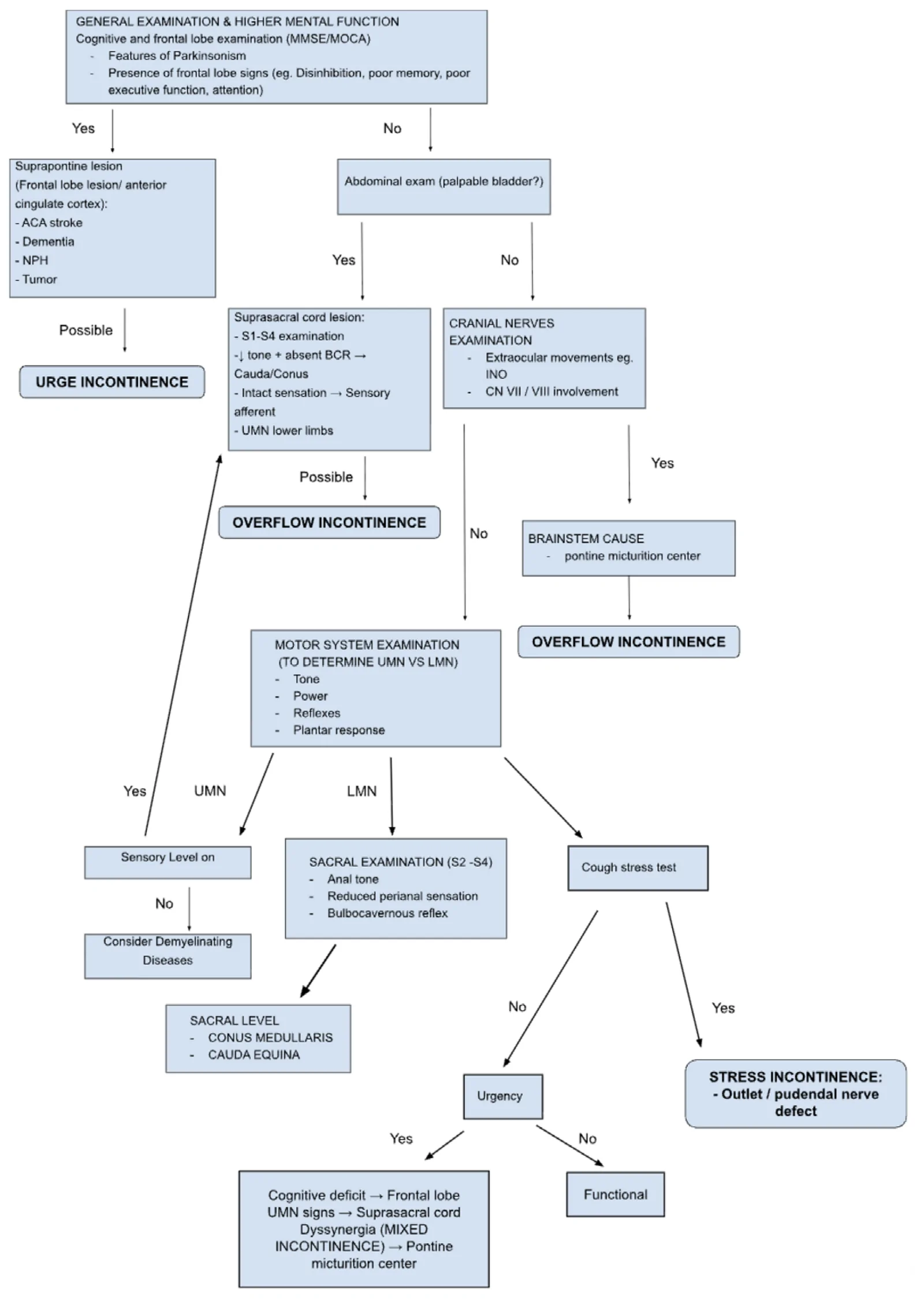
Flowchart for patient examination to identify the cause of incontinence

## Clinical Scenarios

### Case 1

A 70-year-old man presents with involuntary passage of urine. He is able to tell that his bladder is full, but he cannot control the urge to urinate long enough to reach a toilet. His daughter also mentions that he has been falling frequently lately. On examination by Mini Mental Status Examination, he is found to have poor memory and impaired executive functions.

#### Type of Incontinence

Urge incontinence

#### Localisation

The lesion is localised at the frontal lobe or anterior cingulate gyrus (Figure 8).

#### Clinical Relevance

ACA strokes, normal-pressure hydrocephalus and tumours affecting the above regions may present with urge incontinence, along with other symptoms.

### Case 2

A 38-year-old male presents to the emergency department following an injury sustained during heavy lifting, complaining of sudden heaviness in the legs and complete loss of bladder sensation. He complains of passing urine involuntarily at intervals without any urge to do so or pain. He also mentions that he cannot voluntarily delay urination.

On examination, he is found to have a palpably distended bladder. There is also flaccid weakness of the bilateral lower limbs, along with loss of sensation around the anus and genitals, as well as reduced anal tone.

### Type of Incontinence

Overflow incontinence

### Localisation

The combination of afferent (sensory) and efferent (motor) losses confirms a lesion at the sacral cord, conus medullaris or cauda equina

There is a large posterior central disc extrusion at L5–S1 level (Figure 9). This extrusion indents the thecal sac anteriorly, induces severe central canal stenosis, giving rise to the clinical picture above.

### Clinical Relevance

This patient exhibits a failure of the sacral reflex arc. Unlike a suprapontine lesion or a suprasacral spinal cord lesion, this lesion results in an areflexic bladder, leading to overflow.

## Conclusion

The authors hope that this localisation summary and flow chart for causes of urinary incontinence will be helpful in quick localisation and therefore, prompt management of patients with urinary incontinence.

## Figures and Tables

**Figure 1:**
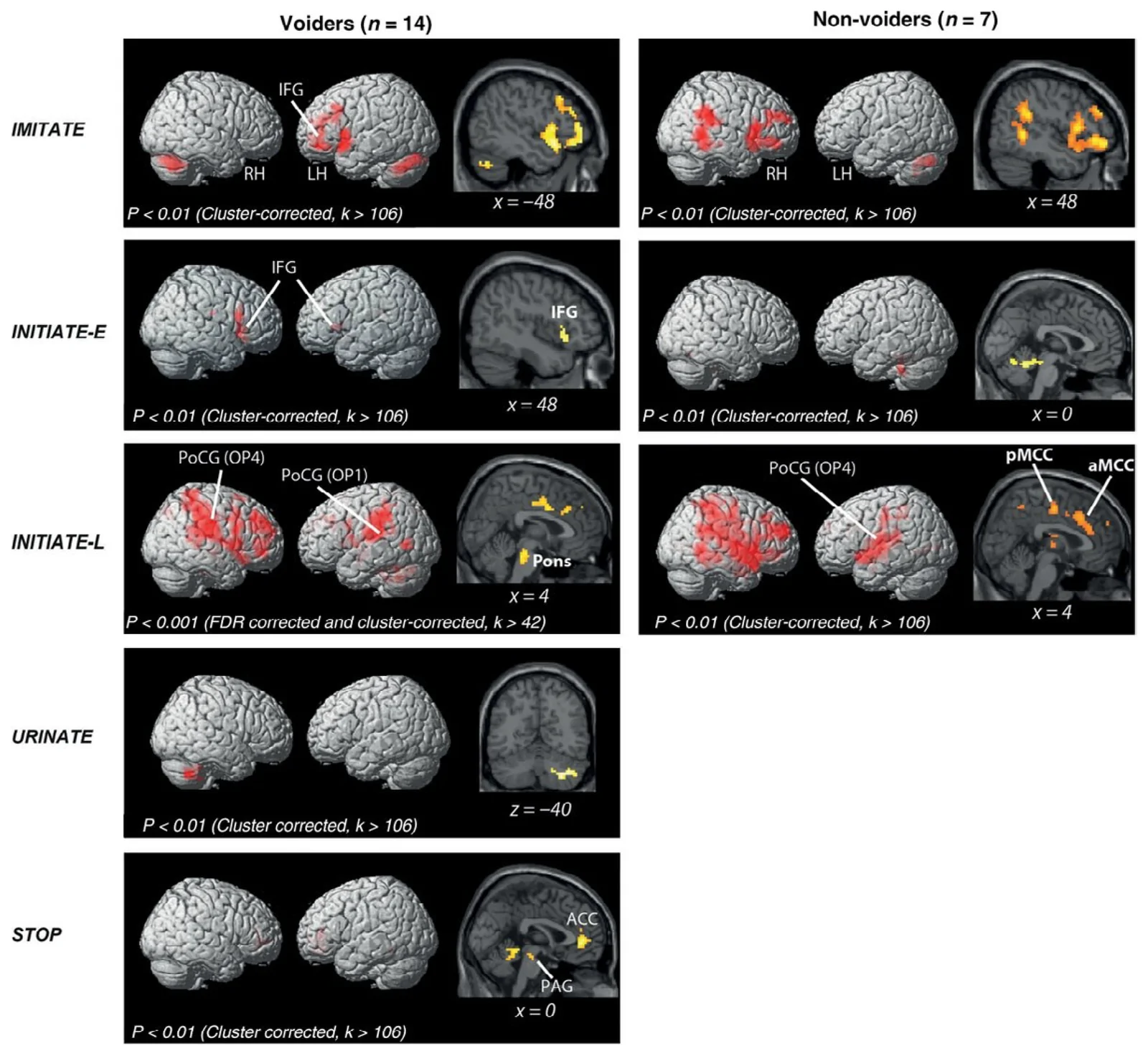
Rendered brain displaying BOLD signal peaks during different conditions (IMITATE, INITIATE-E (early), INITIATE-L (late), URINATE and STOP) compared with REST in subjects who were able (voiders) and unable (non-voiders) to void during scanning (7) ACC = anterior cingulate cortex; LH = left hemisphere; RH = right hemisphere; IFG = inferior frontal gyrus; PAG = periaqueductal grey area; PoCG = postcentral gyrus; OP = parietal operculum; aMCC = anterior midcingulate cortex; pMCC = posterior midcingulate cortex

**Figure 2:**
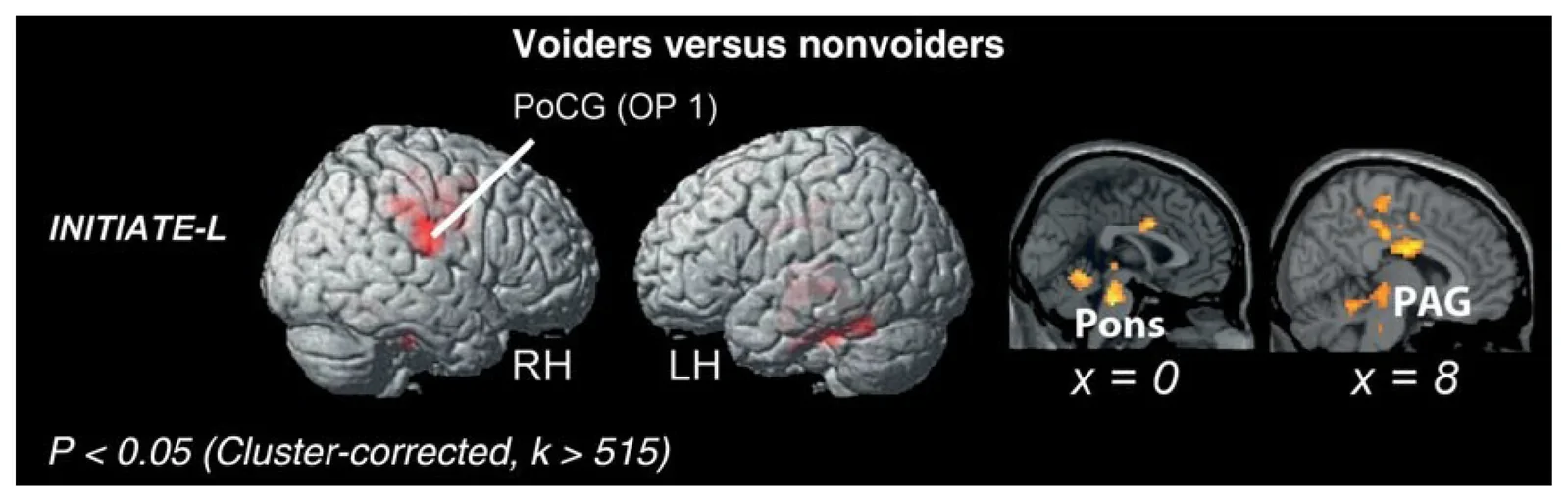
Rendered brain displaying BOLD signal peaks of voiders versus non-voiders (7) LH = left hemisphere; RH = right hemisphere; PoCG = postcentral gyrus; OP = parietal operculum; PAG = periaqueductal grey area

**Figure 3:**
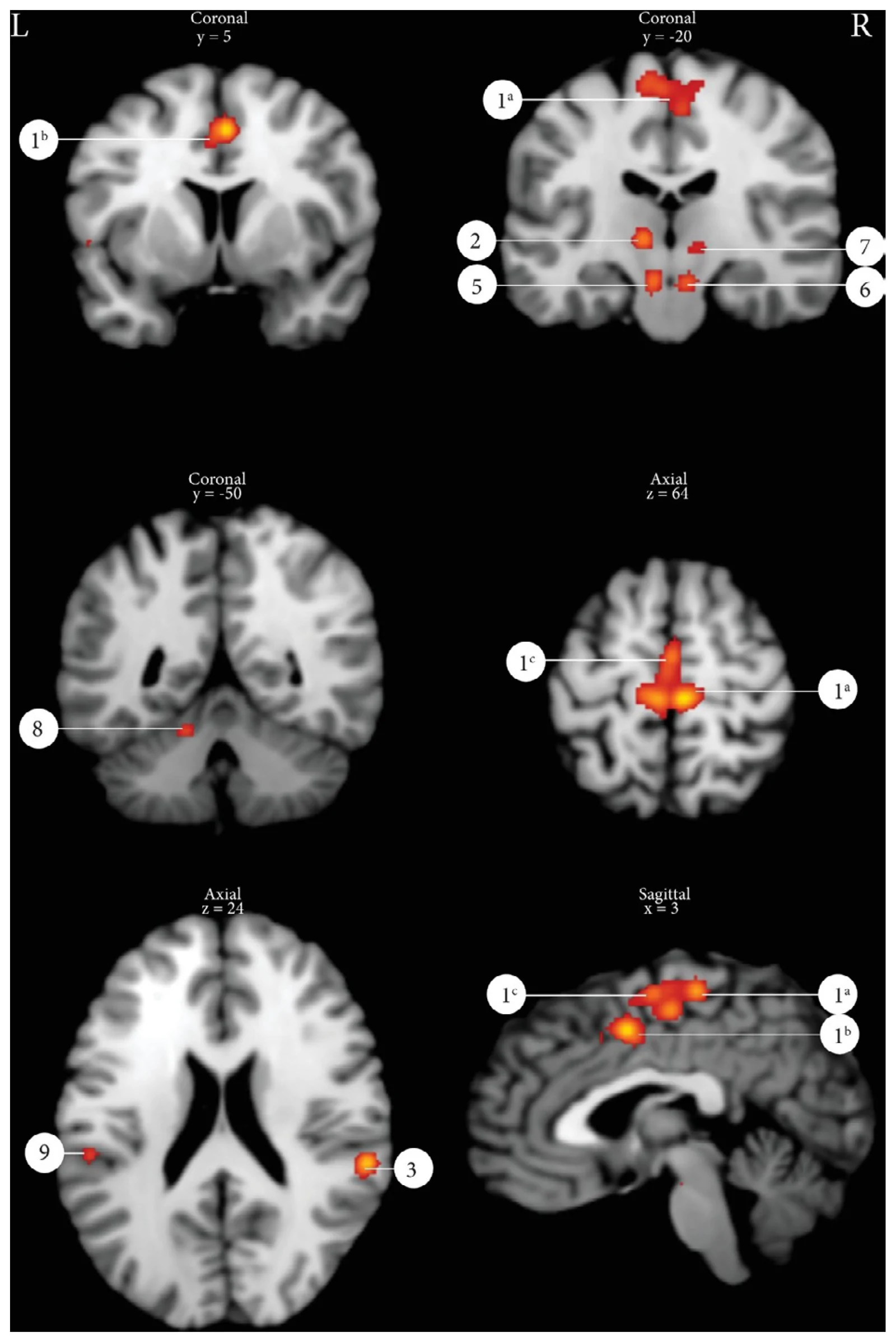
Results of the ALE analysis of PFMC (7) 1a = primary motor cortex; 1b = midcingulate gyrus; 1c = supplementary motor area; 2 = thalamus (left); 3 = supramarginal gyrus right; 5 = substantia nigra; 6 = red nucleus; 7 = thalamus right; 8 = cerebellum left; 9 = supramarginal gyrus left

**Figure 4:**
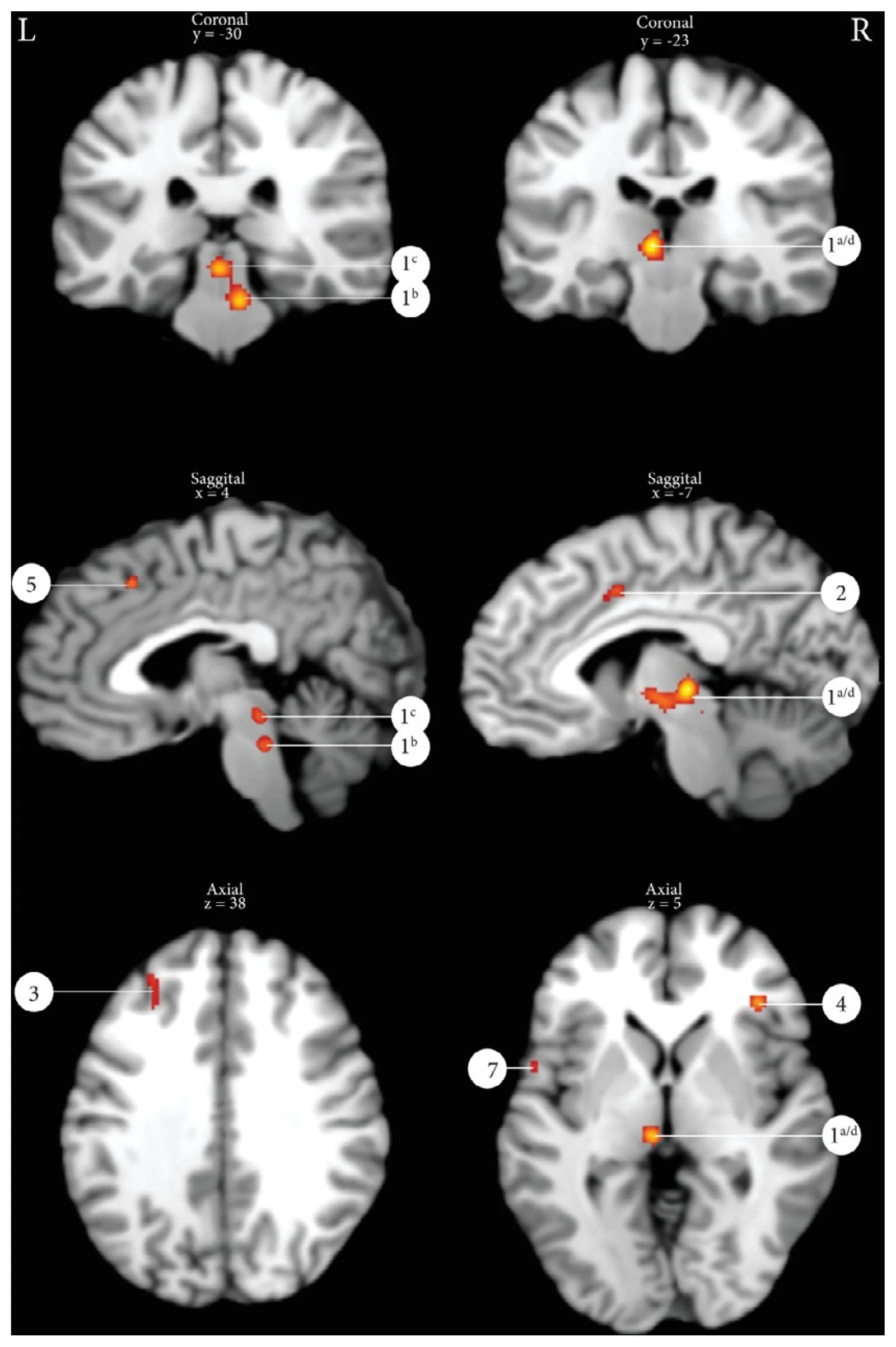
Results of the ALE analysis of micturition (7) 1a/d = thalamus; 1b = pontine micturition centre; 1c = periaqueductal grey area; 2 = cingulate gyrus; 3 = middle frontal gyrus; 4 = insula; 5 = superior frontal gyrus; 6 = ventral pons; 7 = inferior frontal gyrus

**Figure 5:**
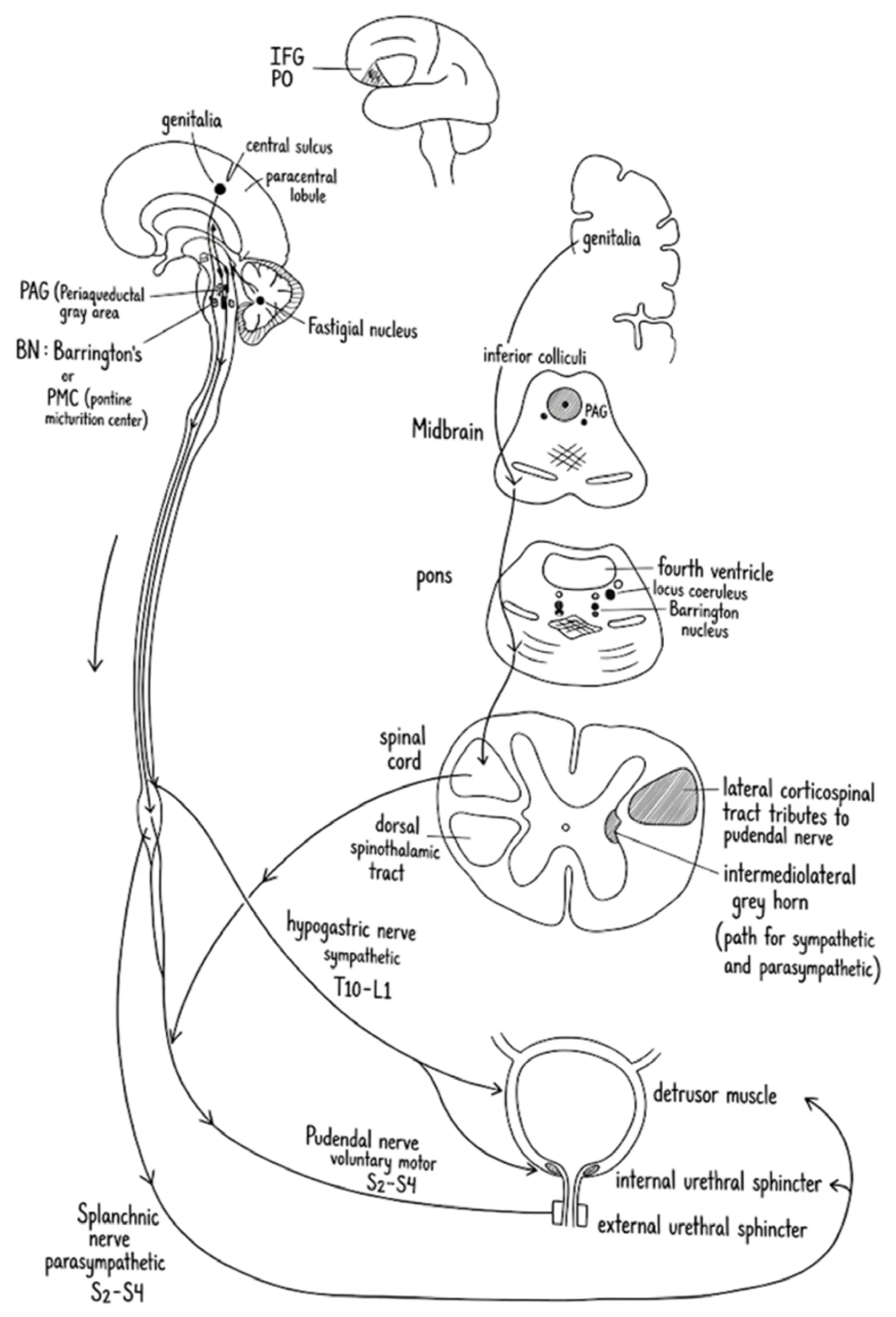
Diagram depicting the anatomical locations of structures involved in micturition

**Figure 8:**
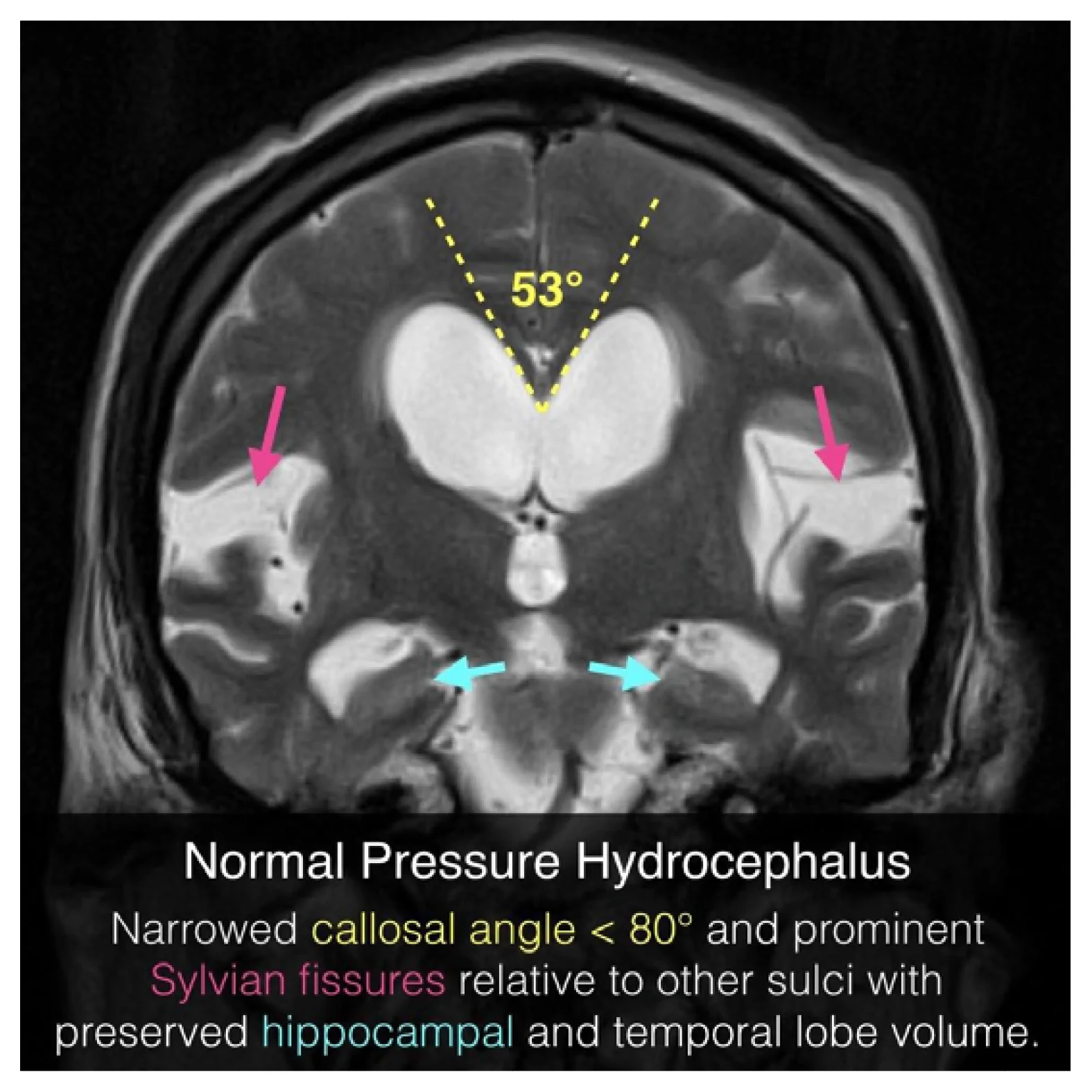
MRI image depicting hydrocephalus Case courtesy of Mostafa Elfeky, Radiopaedia.org, rID: 73685

**Figure 9:**
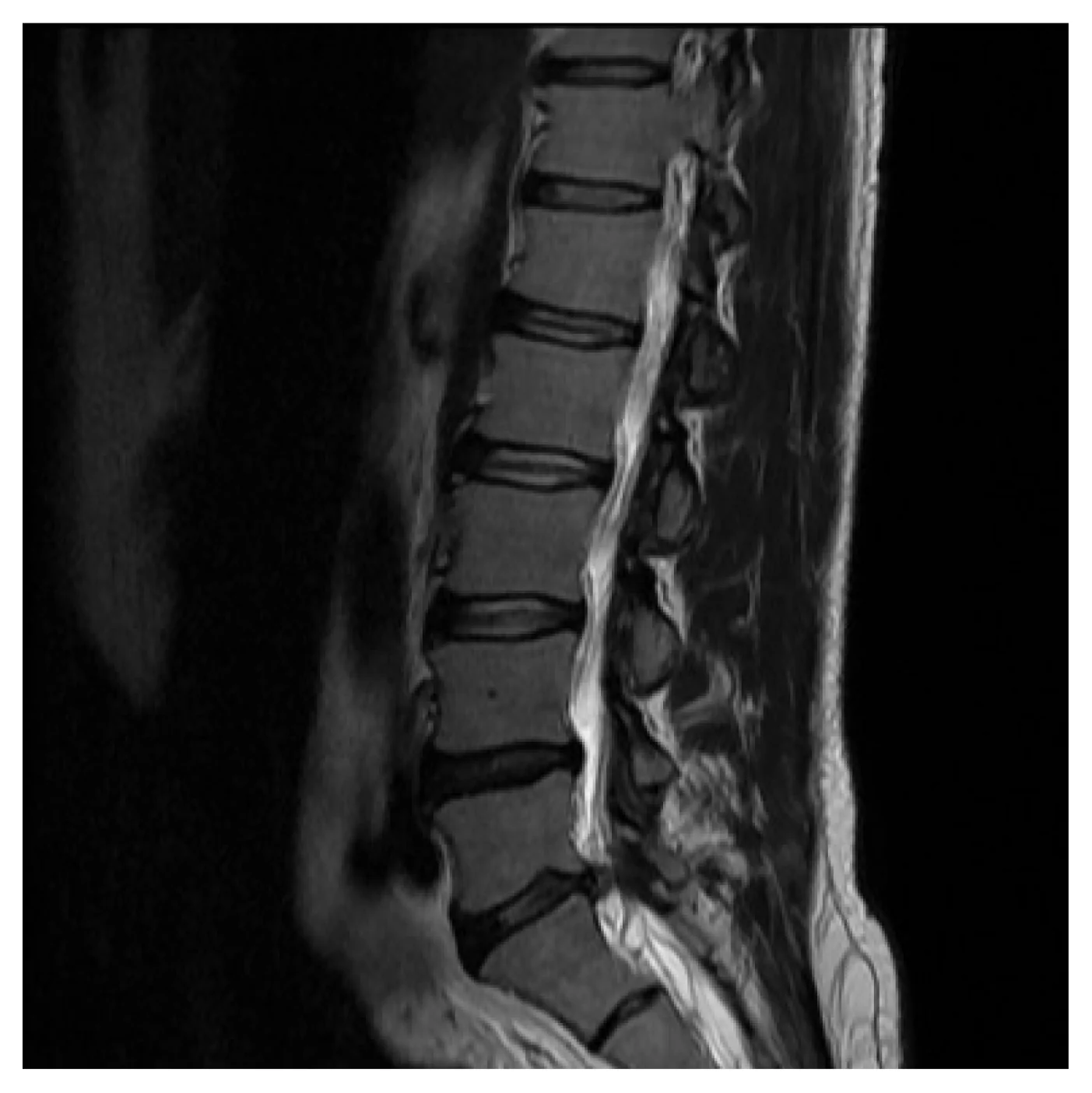
MRI lumbosacral spine sagittal view Case courtesy of Khaloud Alghamdi, Radiopaedia.org, rID: 79069

**Table 1:** Additional history-taking for localisation of the cause of incontinence

Structure involved	Key findings for localisation	Supplementary questions
Prefrontal cortex (suprapontine inhibition)	The patient is aware of the need to void, and voluntary voiding is intact. Only loss of inhibition is present.	“Can you voluntarily start and stop urination when asked?” Confirms intact coordination (rules out pontine lesion) “Do you normally feel it when your bladder is full?” → Confirms an intact afferent pathway “Any difficulty with poor stream or incomplete emptying?” → Absence rules out PMC involvement
Periaqueductal grey area (initiation gate)	Sensory input is intact, and there is no motor deficit. The only deficiency is in initiation.	“Once urine starts, is the flow normal?” → Normal flow excludes pontine dyssynergia “Do you ever void automatically without control?” → No → excludes spinal reflex bladder “Any weakness or sensory loss in limbs?” → No → supports isolated midbrain integration failure
Pontine micturition centre (coordination)	Detrusor–sphincter dyssynergia is present, causing coordination failure.	“Do you need to strain to pass urine?” → Suggests detrusor–sphincter dyssynergia (where is the pathology anatomically?) “Do you normally feel it when your bladder is filling?” → Preserved sensation → excludes afferent lesion (exactly where?) “Do you experience urine leakage without warning?” → No → excludes suprapontine disinhibition
Suprasacral spinal cord (loss of voluntary control)	Reflex voiding is present, but voluntary control is absent.	“Do you still feel that your bladder is full before it empties?” → Often reduced or absent awareness of the need to void “Can you stop urine flow once it starts?” → No → confirms loss of cortical control “Any spasticity or hyperreflexia in the legs?” → Supports suprasacral cord lesion
Sympathetic outflow – storage (T11–L2)	Storage is impaired, but voiding is preserved.	“When you pass urine, can you empty your bladder completely?” → Yes → excludes parasympathetic lesion “Do you experience urine leakage upon coughing or sneezing?” → No → excludes pudendal nerve injury “Any pain or difficulty initiating urination?” → No → excludes PMC involvement
Parasympathetic outflow – voiding (S2–S4)	Detrusor underactivity and overflow incontinence are present.	“Do you experience that the bladder is full or painful?” → Reduced sensation supports sacral involvement “Do you experience continuous urine leakage without urge to urinate?” → Suggests overflow incontinence “Do you have constipation or erectile dysfunction?” → Suggests autonomic sacral involvement
Pudendal nerve (external sphincter control)	External sphincter failure is present, causing stress incontinence.	“Is your urine flow normal when you void?” → Normal → excludes parasympathetic lesion “Do you experience urine leakage at rest?” → No → excludes overflow incontinence “Any numbness around the anus or genitals?” → No → excludes cauda equina
Afferent sensory pathway from the bladder	Sensory loss is present, and reflex motor function is preserved.	“Does urine come out with a normal flow?” → Yes → motor pathways intact “Can you voluntarily delay urination?” → No → loss of sensory feedback “Any diabetes or neuropathy?” → Supports sensory nerve involvement
Sacral cord/cauda equina (complete sacral lesion)	Both afferent and efferent losses are present, causing an areflexic bladder.	“Is there loss of bowel control?” → Confirms sacral involvement “Do you have reduced anal tone?” → requires confirmatory examination “Can you feel that your bladder is full at all?” → No → afferent failure
